# Improvement of Toluene-Sensing Performance of SnO_2_ Nanofibers by Pt Functionalization

**DOI:** 10.3390/s16111857

**Published:** 2016-11-04

**Authors:** Jae-Hun Kim, Zain Ul Abideen, Yifang Zheng, Sang Sub Kim

**Affiliations:** Department of Materials Science and Engineering, Inha University, Incheon 402-751, Korea; kjhhb5331@gmail.com (J.-H.K.); zainulabideen@msn.com (Z.U.A.); zheng.yifang@hotmail.com (Y.Z.)

**Keywords:** Pt, SnO_2_, gas sensor, metal nanoparticle, sensitization

## Abstract

Functionalization of metal nanoparticles (NPs) on oxide materials is a commonly employed technique for enhancing the sensitivity and selectivity of materials for gas sensing applications. In this study, we functionalized electrospinning-synthesized SnO_2_ nanofibers (NFs) with various amounts of Pt NPs to enhance the toluene-sensing properties. In particular, Pt NPs were prepared by deposition of Pt films by sputtering and subsequent heat treatment. Electronic and chemical sensitizations by the Pt NPs were responsible for the improved toluene sensitivity. The best sensing properties were achieved at an optimized amount of Pt NPs, showing a volcano shape in relation to the amount of Pt NPs. The method used in this study is useful for the development of toluene-sensitive and -selective chemiresistive NF-based gas sensors.

## 1. Introduction

Highly sensitive metal oxide-based gas sensors have become increasingly important for monitoring environmental pollution and toxic chemical gases in industry as well as in daily life. The sensitivity of metal oxide-based gas sensors is highly dependent on the specific surface area of the sensing material. Therefore, many attempts have been made to increase the specific surface area of sensing materials in the past few decades [1,2,3,4,5] using one-dimensional structures such as nanowires, nanotubes, nanobelts, and nanofibers (NFs). Among these, NFs have attracted enormous attention due to their high specific surface area. In addition, one-dimensional morphologies are suitable for confined and directional transport of charge carriers. 

In particular, NFs have a unique microstructure, exhibiting nanograins on the surfaces that can further increase the surface area and greatly affect the sensing properties as compared to other one-dimensional structures. Furthermore, the sensitivity of NFs can be easily improved by controlling the size of these surface nanograins [6,7], the use of composite NFs [8,9], and the functionalization of catalytic metal nanoparticles (NPs) [10,11]. Functionalization or decoration with metal NPs is a generally accepted and effective route to enhance the gas sensing properties of metal oxides [12,13] by electronic and chemical sensitizations. 

Among the various methods for fabricating NFs, electrospinning is an efficient, relatively easy, and novel technique to produce NFs from viscous solutions. Due to the high efficiency, good control over the processing parameters, and suitable characteristics of the resulting NFs, electrospinning is one of the main fabrication techniques for NFs [14]. To date, NFs from many functional materials have been successfully synthesized and investigated for gas sensing applications.

There is ample literature regarding the functionalization of SnO_2_, a well-known n-type semiconductor material that has been widely studied for gas sensing applications, and the improvement in its sensitivity and selectivity upon loading or decorating various noble metals such as Pd [15], Au [11], Ag [16], Pt [17], and various oxide and non-oxide materials [18,19,20]. Additionally, a variety of methods for functionalization of such noble metals have been employed as shown in Table S1 [21,22,23,24,25,26,27,28]. The role of metal nanoparticles (NPs) in gas sensing properties is well established as chemical and electronic sensitizations. In addition, the amount of the metal NPs used to functionalize the surface greatly influences the sensing properties of the metal oxides; therefore, optimization of this factor is highly desirable.

One of the most emerging applications of chemiresistive-type gas sensors is non-invasive disease diagnostics through the detection of specific volatile organic compounds or gaseous biomarkers [29,30,31]. Toluene (C_7_H_8_) gas is a recognized biomarker for diagnosing lung cancer [32,33]. According to the earlier investigation [34], functionalization of Pt NPs resulted in enhanced sensitive and selective toluene-sensing behavior of the core-shell nanowires. A more recent work [35] revealed the special role of Pt in relation to the toluene sensing based on Density Functional Theory (DFT) calculations. In the previous work [10], Pt-loaded SnO_2_ NFs were synthesized and tested, in which Pt NPs were synthesized in a separate process and mixed with the electrospinning solution. In order to expedite the use of Pt-functionalized NF-based sensors, various methods of functionalizing Pt NPs and their toluene-sensing properties needs to be investigated. On the other hand, Pt is one of precious metals, the use of which is costly. Therefore, finding an alternative element that is earth abundant is significant with regards to real application and mass-production. 

In this study, SnO_2_ NFs, synthesized by electrospinning, were functionalized with different amounts of Pt NPs. The amount of Pt NPs was controlled by changing the thickness of the sputter-deposited Pt layers on SnO_2_ NFs. After a thermal treatment, Pt layers were disintegrated into isolated islands, resulting in functionalization of Pt NPs on SnO_2_ NFs. Although there are many earlier investigations regarding Pt-loaded or decorated SnO_2_ sensing materials [10,22], the SnO_2_ NFs with Pt NPs have rarely been investigated. Furthermore, the optimization of the Pt amount has never been attempted. In this work, an optimized amount of Pt NPs showed the best sensing properties.

## 2. Materials and Methods

The procedure used to synthesize Pt-functionalized SnO_2_ NFs is as follows. An aqueous solution of polyvinyl acetate (PVAc, Mw = 850,000, Sigma-Aldrich Corp, Cream Ridge, NJ, USA) was prepared in a mixed solvent (volume ratio 1:1) of ethanol (anhydrous, 99.5%, Sigma-Aldrich Corp, Cream Ridge, NJ, USA) and dimethylformamide (DMF, 99.8%, Sigma-Aldrich Corp, Cream Ridge, NJ, USA) and continuously stirred for 4 h at room temperature. Subsequently, 12.3 wt % tin(II) chloride dihydrate (SnCl_2_·2H_2_O, Sigma-Aldrich Corp, Cream Ridge, NJ, USA) was added to the prepared solution and continuously stirred for 12 h.

The SnO_2_ NFs were prepared using an electrospinning process. The prepared viscous precursor solution was loaded into a syringe equipped with a 21-guage needle. A positive voltage of 15 kV was applied to the needle tip and the metal collector was grounded. The feed rate of the solution was 0.03 mL/h and the distance between the needle tip and the collector was fixed at 20 cm. The electrospun NFs were collected on SiO_2_-grown Si wafers that had been placed on the metal collector. The prepared SnO_2_ NFs were then calcined at 650 °C for 2 h with a heating rate of 5 °C/min.

The SnO_2_ NFs were functionalized with Pt NPs according to the following procedure. First, Pt thin films of different thicknesses (3, 5, 10, 15, and 20 nm) were deposited using magnetron sputtering. The magnetron sputtering conditions were as follows; input power 30 W, target diameter 50 mm, deposition temperature 25 °C, Ar gas pressure 2.65 Pa, target-to-substrate distance 100 mm. The thickness of Pt films was controlled by changing the deposition time. The relationship between the deposition time and Pt thickness was established by measuring the thickness of Pt films deposited on Si (100) substrates under the same sputtering conditions used in this work. Subsequently, the as-deposited samples were heat-treated at 500 °C in air for 0.5 h. During this time, the Pt layers transformed into Pt NPs through self-arrangement. The size of the Pt NPs increased with increasing thickness of the sputtered Pt layer.

Microstructural and morphological analyses of the Pt-functionalized SnO_2_ NFs were carried out using field-emission scanning electron microscopy (FE-SEM). The compositional analysis was performed using energy dispersive spectroscopy (EDS). Crystal structures and detailed microstructures were investigated by X-ray diffraction (XRD) and transmission electron microscopy (TEM), respectively. For the gas sensing measurements, a layer of Ti (thickness of 50 nm) followed by a Pt layer (thickness of 200 nm) were sputter deposited to make electrodes over the calcined Pt-functionalized SnO_2_ NFs using interdigital electrode masks. The interdigital electrode pattern consisted of eight fingers with dimensions of 7 mm length and 0.5 mm width, while 150 μm spacing was used to deposit the Ti/Pt double-layer electrode. The details of the fabrication process for the NF sensor devices are described in earlier reports [8,36]. The properties of the sensors were evaluated at an optimized temperature of 300 °C with C_7_H_8_, using a gas dilution system. The gas flow was controlled by mixing the target gas with dry air using accurate mass flow controllers. The sensing system was electrically connected to a measurement system (Keithley 2400) and interfaced to a computer. The sensors were placed in a horizontal-type tube furnace and the temperature was controlled by changing the mixing ratio of the dry air-balanced target gas and the dry air through accurate mass flow controllers. A detailed experimental procedure including a schematic for the sensing measurement system is provided in our earlier report [9]. 

In order to investigate the cross sensitivity, other reducing gases such as benzene (C_6_H_6_) and carbon dioxide (CO_2_) were also tested. The sensor response was estimated by the relationship R_a_/R_g_, where R_a_ represents the resistance of the sensor in the absence of the target gas and R_g_ is the resistance in the presence of the target gas. 

## 3. Results and Discussion

**Figure 1:**
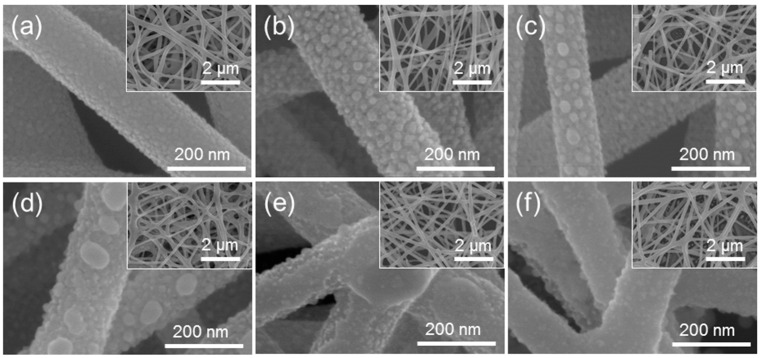
FE-SEM images of (**a**) pure SnO_2_ NFs, and SnO_2_ NFs functionalized with Pt NPs containing (**b**) 0.2 at.%; (**c**) 0.3 at.%; (**d**) 1.2 at.%; (**e**) 2.0 at.%; and (**f**) 5.4 at.% Pt; Pt films of 3, 5, 10, 15, and 20 nm, respectively were deposited and subsequently heat-treated. The insets are the corresponding low-magnification FE-SEM images of SnO_2_ NFs functionalized with NPs.

Figure 1 shows the microstructures of the Pt-functionalized SnO_2_ NFs observed using FE-SEM. Figure 1a shows the typical microstructure of pure SnO_2_ NFs for comparison. Figure 1b–f show SnO_2_ NFs onto which various amounts of Pt were deposited (3, 5, 10, 15, and 20 nm, respectively) and subsequently heat-treated. The average diameter of the fibers was ~165 nm. It is obvious from these images that the Pt layers broke into small clusters or islands during the heat treatment process. The thinner layers formed many smaller Pt clusters while the thicker layers broke into fewer, but larger clusters. In the case of the 15 and 20 nm layers (Figure 1e,f, respectively), the islands were connected and remained almost as a continuous thin layer covering most parts of the NFs’ surface. This is because the thin layers easily break and are converted into smaller islands, while it is more difficult to break the thicker layers under the same heat treatment conditions. It can also be noted that the varying amounts of deposited Pt did not affect the size or shape of the NFs and nanograins. Overall, the NFs were uniformly and randomly distributed over the Si substrates, as shown in the insets, the corresponding low-magnification FE-SEM images.

**Figure 2:**
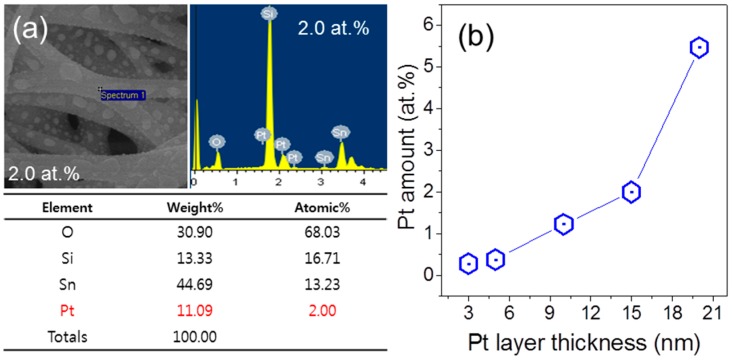
(**a**) Elemental analysis of 2.0 at.% Pt-functionalized SnO_2_ NFs using EDS; (**b**) Relationship between Pt amount and Pt layer thickness.

In order to confirm the presence of Pt NPs, elemental analyses were carried out using EDS as shown in Figure 2a and Figure S1. From Figure 1, in conjunction with Figure 2a and Figure S1, we can conclude that we were successful in synthesizing the SnO_2_ NFs with different amounts of Pt NPs. The amount of Pt NPs formed after the heat treatment increased linearly with increasing thickness of the Pt layers deposited by sputtering, as summarized in Figure 2b. The linearity suggests the easy control of the amount of Pt NPs by changing the thickness of Pt layers. Quantitative analysis by EDS usually has some degree of uncertainty. In Figure S2, elemental analysis, including errors in quantitative calculation by EDS of SnO_2_ NFs functionalized with Pt NPs, is provided. In spite of the small errors, the linear relationship between the amount of Pt NPs and thickness of Pt layers is remained the same.

**Figure 3:**
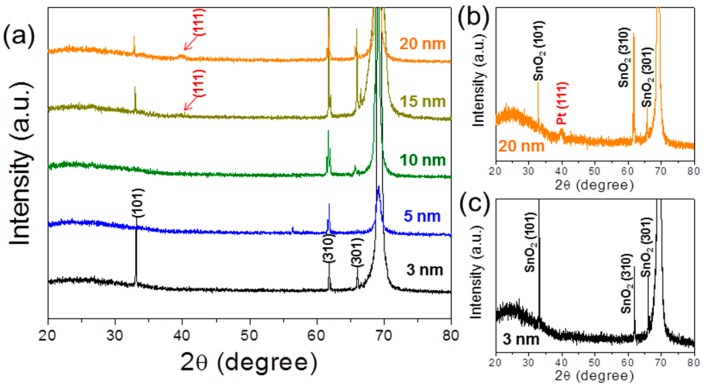
(**a**) XRD patterns for Pt-SnO_2_ NFs with various Pt layer thicknesses: (**b**) 20 nm-thick, and (**c**) 3 nm-thick Pt layers.

Crystal structures of the SnO_2_ NFs functionalized with Pt NPs were investigated by using XRD, and the results are shown in Figure 3. As is evidently shown, the samples coated with Pt layers over 15 nm in thickness reveal Pt (111) peaks.

**Figure 4:**
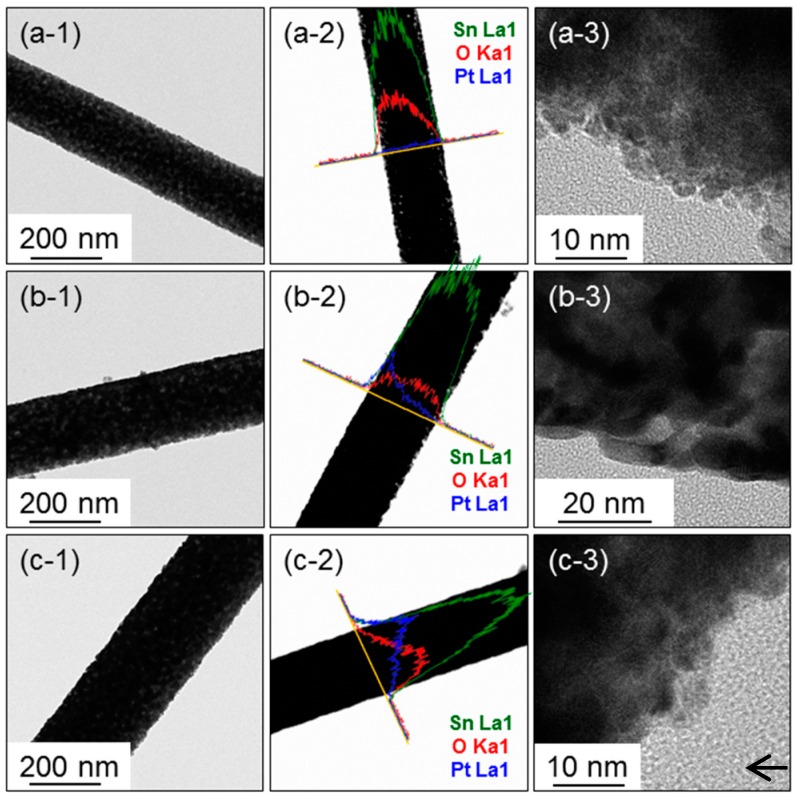
TEM results taken from Pt-SnO_2_ Nanowires (NWs) with 3 nm-thick Pt layer: (**a-1**) low magnification TEM image, (**a-2**) EDS elemental line profiles, and (**a-3**) a high-resolution TEM image; 10 nm-thick Pt layer: (**b-1**) a low magnification TEM image, (**b-2**) EDS elemental line profiles, and (**b-3**) a high-resolution TEM image; 20 nm-thick Pt layer: (**c-1**) a low magnification TEM image, (**c-2**) EDS elemental line profiles, and (**c-3**) a high-resolution TEM image.

The microstructure of the SnO_2_ NFs functionalized with Pt NPs was further investigated by using TEM. As shown in Figure 4, individual NFs are quite uniform in diameter. The elemental line mappings, displayed in Figure 4(b-2,c-2), demonstrate the presence of a Pt element. In Figure 4(c-3), the arrow indicates the Pt NP.

**Figure 5:**
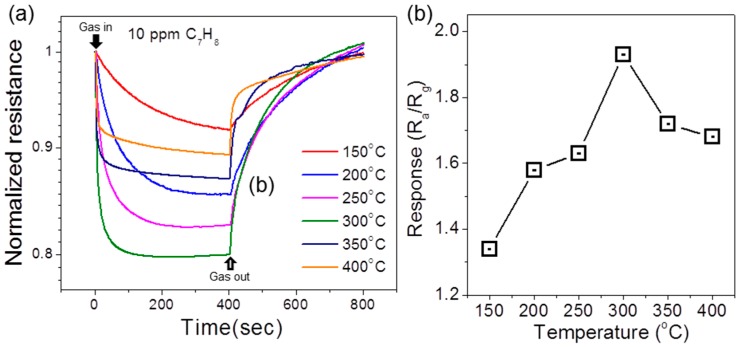
(**a**) Resistance curves of pure SnO_2_ NFs exposed to 10 ppm C_7_H_8_ at various temperatures; (**b**) Responses of pure SnO_2_ as a function of operating temperature.

The optimal temperature was investigated by testing the sensing properties of pure SnO_2_ NFs at various temperatures (150 to 400 °C) using 10 ppm of C_7_H_8_. Figure 5a shows the resistance curves obtained at various temperatures. The response was summarized in Figure 5b, indicating the optimal temperature was found to be 300 °C. On the basis of the result, hereafter the sensing properties were investigated at that temperature. The resistance of SnO_2_ NFs decreases upon the introduction of C_7_H_8_ gas and recovers to its original value when the gas is removed, as shown in Figure 5a.

The sensing mechanism of SnO_2_ NFs can be explained within the framework of n-type semiconductors, in which the majority of the charge carriers are electrons. In ambient air, oxygen interacts with the surface, diffuses through the grain boundaries of the nanograins in individual NFs, and becomes ionized by extracting electrons from the conduction band of SnO_2_. The loss of electrons due to the ionization of the oxygen gas develops an electron-depleted region underneath the interface and upward band bending at the grain boundaries, increasing the potential barriers to the flow of electrons across the grain boundaries. The potential barriers and the depletion region are significantly suppressed when C_7_H_8_ is introduced as it interacts with adsorbed oxygen species, making volatile compounds, eventually releasing captured electrons to the conduction band of SnO_2_. This is the main source of resistance modulation in SnO_2_ NFs.

**Figure 6:**
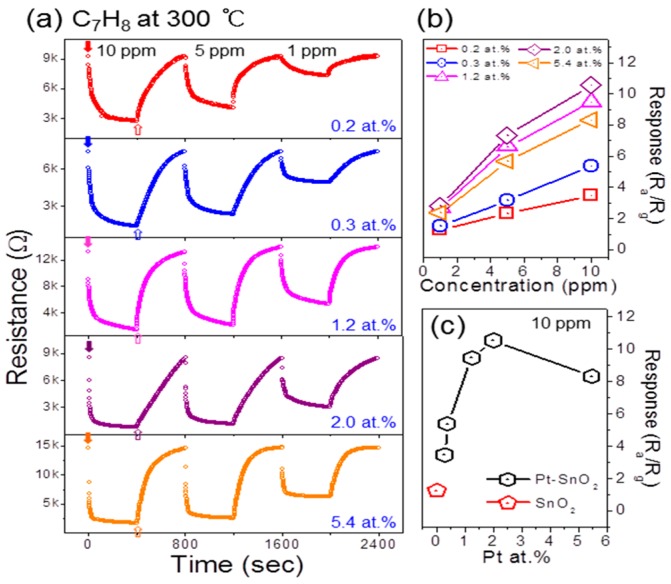
(**a**) Resistance curves of Pt-functionalized SnO_2_ NFs exposed to C_7_H_8_ at 300 °C; (**b**) Summarized responses for various C_7_H_8_ concentrations; (**c**) Responses to 10 ppm C_7_H_8_ with regard to the amount of Pt functionalization. The response of pure SnO_2_ NFs to 10 ppm C_7_H_8_ is included for comparison.

The sensing performances and the effect of the Pt amount were then evaluated at 300 °C using various concentrations of C_7_H_8_ gas. Figure 6a shows the typical resistance curves and responses of the SnO_2_ NFs with varying amounts of Pt NPs to low concentrations (1, 5, and 10 ppm) of C_7_H_8_ gas. The highest response was observed from the NFs with 2.0 at.% Pt, as shown in Figure 6b, where the responses are plotted as a function of gas concentration.

Figure 6c summarizes the responses of Pt-functionalized SnO_2_ NFs to 10 ppm C_7_H_8_ gas as a function of Pt amount. The response of pure SnO_2_ has been included for comparison. It is evident that the attachment of Pt NPs significantly enhanced the sensitivity of SnO_2_ NFs. Moreover, Figure 6c shows a bell-shaped curve as a function of the Pt NP amount where there is an initial improvement in the response with smaller amounts of Pt and then a deterioration with larger amounts. The possible explanation of this bell-shaped behavior as a function of the amount of Pt will be explained at a later part of this section.

In contrast to the pure SnO_2_ NFs, two mechanisms can be considered to be responsible for the improved gas sensing properties of Pt-functionalized SnO_2_ NFs; (1) electronic sensitization and (2) chemical sensitization of Pt NPs [15,37,38,39]. In electronic sensitization, Pt NPs interact with SnO_2_ NFs electronically by acting as electron acceptors due to a difference in the work function, which increases the depth of the electron-depletion region and the heights of the neighboring potential barriers across the grains. In this way, the metal functionalized oxides become more sensitive to the environmental changes [15]. Whereas in the catalytic or spillover effect, Pt NPs can increase the interaction by providing more active sites for the dissociation of gas molecules due to their highly conductive nature [40]. There is an increase in the number and speed of electrons transferred to the SnO_2_ grains as a result of the spillover effect. The improved sensitivity of Pt-functionalized SnO_2_ NFs originates from a combination of the electronic and chemical sensitization mechanisms and involves multiple factors, including the spillover effect, the chemisorption and dissociation of the gas molecules, the kinetics of the electron transfer, and the net effect of location and chemical states of the metal NPs. However, the electronic sensitization is likely to play a more important role than chemical sensitization in the overall enhancement of the sensitivity due to the change in its oxidation states [12,15,41].

The bell-shaped behavior of metal-functionalized oxides as a function of the amount of metal NPs (Figure 6c) is common and often reported in the literature [16,42,43,44,45,46]. The sensitivity of functionalized oxides is greatly influenced by the loading concentration and position of the metal NPs. This behavior can be understood in terms of the surface coverage of the metal NPs (Pt) on the oxide material (SnO_2_). The sensitization effect will be marginal at both insufficient and excessive surface coverage of metal NPs, due to the small number of NPs participating in the process, and steric hindrance, respectively. Over a certain surface coverage, the metal NPs may partially or completely connect with each other, covering the surface of the oxide (reducing the interaction and reaction activity of the target gas with the oxide surface) and causing the electrons to flow in the metallic layer owing to its high conductivity. This agglomeration of metal NPs is evident from Figure 1f. Therefore, the amount of Pt NPs must be optimized for better enhancement of the gas sensing at the optimal operating temperature.

**Figure 7:**
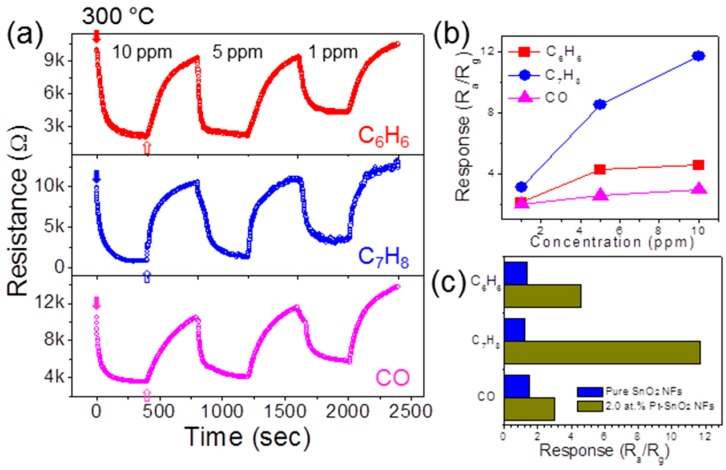
(**a**) Resistance curves of 2.0 at.% Pt-functionalized SnO_2_ NFs for other reducing gases at 300 °C; (**b**) Summarized responses; (**c**) Comparison of the responses of 2.0 at.% Pt-functionalized SnO_2_ NFs with those of pure SnO_2_ NFs for 10 ppm reducing gases such as C_7_H_8_, C_6_H_6_, and CO.

The selectivity, which is one of the major objectives of the current development of chemiresistive-type gas sensors, was investigated by exposing the sensors to such reducing gases as CO and C_6_H_6_. The tested gas concentration was set to 1, 5, and 10 ppm. The resistance curves are shown in Figure 7a and the responses are summarized in Figure 7b. It can be seen that the sensors showed the highest response to C_7_H_8_. The responses to the other reducing gases were significantly lower in comparison to that of C_7_H_8_.

In Figure 7c, the responses of 2.0 at.% Pt-functionalized SnO_2_ NFs are compared with pure SnO_2_ NFs for 10 ppm gases at 300 °C. This indicates the high catalytic effect of Pt NPs toward C_7_H_8_. Similar selective and enhanced catalysis behaviors of C_7_H_8_ by Pt NPs have been reported in our earlier studies [10,35], which show that the Pt NPs exhibit efficient catalytic activity for enhancing the diffusion and interaction with C_7_H_8_ gas compared to other reducing gases.

## 4. Conclusions

Pt-functionalized SnO_2_ NF gas sensors with different amounts of Pt NPs were synthesized. The effects of the Pt concentration and operation temperature on the gas-sensing properties were investigated. It was observed that the Pt functionalization greatly enhanced the sensitivity of the SnO_2_ NFs. This was attributed to the synergic effect by the electronic and chemical sensitizations originated from the work function difference between Pt and SnO_2_ and the catalytic behavior of the Pt NPs, respectively. A bell-shaped behavior in the sensing curve was observed as a function of the loading amount of Pt NPs, with a maximum response at 2.0 at.% Pt. The results show that the optimization of the loading amount of metal NPs is the major factor influencing the sensitivity of metal-functionalized oxide-based gas sensors.

## Supplementary Materials

The following are available online at http://www.mdpi.com/1424-8220/16/11/1857/s1. Table S1: Various approaches for functionalization of tin oxide nanostructures and their responses; Figure S1: Elemental analysis by EDS of SnO_2_ NFs functionalized with Pt NPs containing (a) 0.2 at.%, (b) 0.3 at.%, (c) 1.2 at.%, (d) 2.0 at.%, and (e) 5.4 at.% Pt. Figure S2: Elemental analysis including errors in quantitative calculation by EDS of SnO_2_ NFs functionalized with Pt NPs containing (a) 0.2 at.%, (b) 0.3 at.%, (c) 1.2 at.%, (d) 2.0 at.%, and (e) 5.4 at.% Pt.
