# Supplementary material for: Improvement of Toluene-Sensing Performance of SnO2 Nanofibers by Pt Functionalization
Source: Sensors (Basel). 2016 Nov 4;16(11):1857. doi: 10.3390/s16111857 (PMC5134516; doi:10.3390/s16111857)
Supplement: Supplementary file 1 [file sensors-16-01857-s001.pdf]

Supplementary Materials: Improvement of Toluene-Sensing Performance of SnO_2 Nanofibers by Pt Functionalization

Jae-Hun Kim, Zain Ul Abideen, Yifang Zheng and Sang Sub Kim

Table S1. Various approaches for functionalization of tin oxide nanostructures and their responses.

SnO_2 Morphology	Synthesis Method	Type of Gas	Temperature (°C)	Response	Reference
Cu-doped SnO_2 spheres	Hydrothermal	H_2S (300 ppm)	300	40	[21]
Pt/SnO_2 catalysts	Wet impregnation	CO	150	>50	[22]
Pd/SnO_2 nanostructures	Microwave assisted hydrothermal	CO (200 ppm)	200	7	[23]
Pd-SnO_2 nanowires	Sol-gel method	CO (2000 ppm)	275	7	[24]
CuO/SnO_2 composite	Catalytic oxidation	C_6H_6 (50 ppm)	280	6.3	[25]
V_2O_5/SnO_2 composite	Non-aqueous synthesis	C_6H_6 (50 ppm)	270	<5.5	[26]
TiO_2/SnO_2 nanosheets	Hydrothermal process	$(C_2H_5)_3N$ (100 ppm)	260	52.3	[27]
Pt-SnO_2 hierarchical nanocomposites	Dipping and precipitation method	C_2H_5OH (100 ppm)	100	11.2	[28]
Pt NPs-functionalized SnO_2 nanofibers	Electrospinning and sputtering	C_7H_8 (10 ppm)	300	11.9	Present work

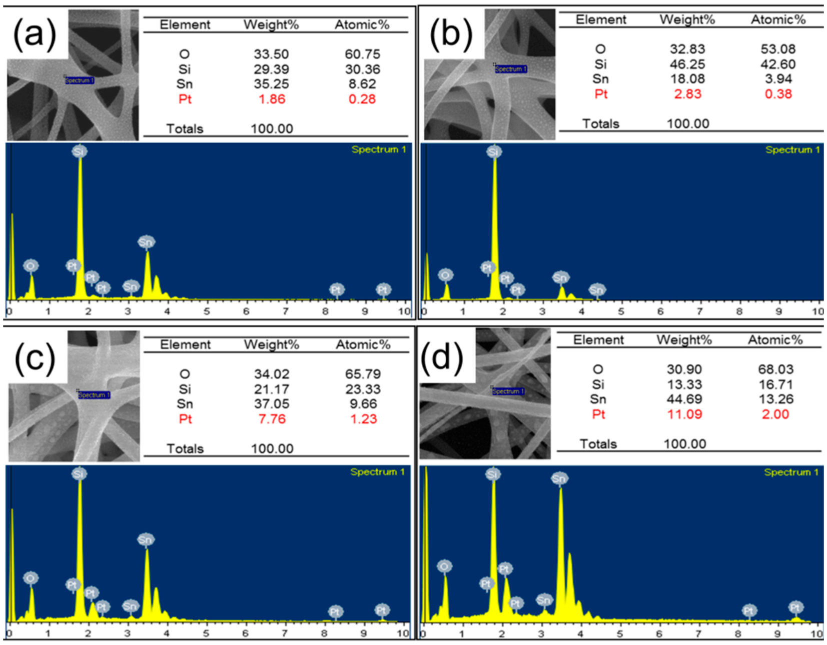

Figure S1. *Cont.*

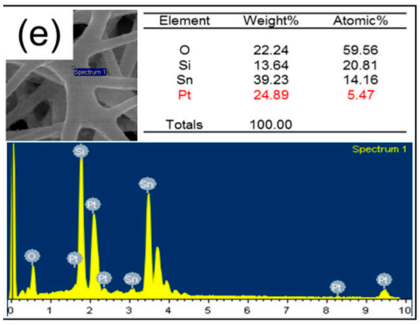

Figure S1. Elemental analysis by EDS of SnO_2 NFs functionalized with Pt NPs containing (**a**) 0.2 at.%; (**b**) 0.3 at.%; (**c**) 1.2 at.%; (**d**) 2.0 at.%; and (**e**) 5.4 at.% Pt.

0.2 at.% (**a**)

Element	Weight%	Atomic%
O K	33.50 +/- 0.13	60.75
Si K	29.39 +/- 0.33	30.36
Sn L	35.25 +/- 0.32	8.62
Pt M	1.86 +/- 0.19	0.28
Totals	100.00	

0.3 at.% (**b**)

Element	Weight%	Atomic%
O K	32.83 +/- 0.13	53.08
Si K	46.25 +/- 0.28	42.60
Sn L	18.08 +/- 0.32	3.94
Pt M	2.83 +/- 0.20	0.38
Totals	100.00	

1.2 at.% (**c**)

Element	Weight%	Atomic%
O K	34.02 +/- 0.11	65.79
Si K	21.17 +/- 0.09	23.33
Sn L	37.05 +/- 0.26	9.66
Pt M	7.76 +/- 0.23	1.23
Totals	100.00	

2.0 at.% (**d**)

Element	Weight%	Atomic%
O K	30.90 +/- 0.13	68.03
Si K	13.33 +/- 0.33	16.71
Sn L	44.69 +/- 0.37	13.26
Pt M	11.09 +/-0.30	2.00
Totals	100.00	

5.4 at.% (**e**)

Element	Weight%	Atomic%
O K	22.24 +/- 0.12	59.56
Si K	13.64 +/- 0.21	20.81
Sn L	39.23 +/- 0.40	14.16
Pt M	24.89 +/- 0.38	5.47
Totals	100.00	

$$at.\% = \frac{\left(\frac{wt.\%}{atomic\ weight}\right)}{total\ atomic\ mole} \times 100$$

Figure S2. Elemental analysis including errors in quantitative calculation by EDS of SnO_2 NFs functionalized with Pt NPs containing (**a**) 0.2 at.%; (**b**) 0.3 at.%; (**c**) 1.2 at.%; (**d**) 2.0 at.%; and (**e**) 5.4 at.% Pt.
